# Cross-sectional Analysis of Food Insecurity and Frequent Emergency Department Use

**DOI:** 10.5811/westjem.2021.3.50981

**Published:** 2021-07-14

**Authors:** Alex Estrella, Joy Scheidell, Maria Khan, Donna Castelblanco, Tod Mijanovich, David C. Lee, Lillian Gelberg, Kelly M. Doran

**Affiliations:** * UMMS-Baystate, Department of Emergency Medicine, Springfield, Massachusetts; † New York University School of Medicine, NYU Langone Health, Department of Population Health, New York, New York; ‡ New York City Department of Health and Mental Hygiene, New York, New York; § New York University Steinhardt School of Culture, Education, and Human Development, Department of Applied Statistics, Social Science, and Humanities, New York, New York; ¶ New York University School of Medicine, Departments of Emergency Medicine and Population Health, New York, New York; || David Geffen School of Medicine at UCLA, Department of Family Medicine, Los Angeles, California; # UCLA Fielding School of Public Health, Department of Health Policy and Management, Los Angeles, California; ** VA Greater Los Angeles Healthcare System, Office of Healthcare Transformation and Innovation, Los Angeles, California

## Abstract

**Introduction:**

Emergency department (ED) patients have higher than average levels of food insecurity. We examined the association between multiple measures of food insecurity and frequent ED use in a random sample of ED patients.

**Methods:**

We completed survey questionnaires with randomly sampled adult patients from an urban public hospital ED (n = 2,312). We assessed food insecurity using four questions from the United States Department of Agriculture Household Food Security Survey. The primary independent variable was any food insecurity, defined as an affirmative response to any of the four items. Frequent ED use was defined as self-report of ≥4 ED visits in the past year. We examined the relationship between patient food insecurity and frequent ED use using bivariate and multivariable analyses and examined possible mediation by anxiety/depression and overall health status.

**Results:**

One-third (30.9%) of study participants reported frequent ED use, and half (50.8%) reported any food insecurity. Prevalence of food insecurity was higher among frequent vs. non-frequent ED users, 62.8% vs 45.4% (P <0.001). After controlling for potential confounders, food insecurity remained significantly associated with frequent ED use (adjusted odds ratio 1.48, 95% confidence interval, 1.20–1.83). This observed association was partially attenuated when anxiety/depression and overall health status were added to models.

**Conclusion:**

The high observed prevalence of food insecurity suggests that efforts to improve care of ED patients should assess and address this need. Further research is needed to assess whether addressing food insecurity may play an important role in efforts to reduce frequent ED use for some patients.

## INTRODUCTION

Even before the coronavirus disease (COVID-19) pandemic, food insecurity affected over 10% of United States households, including nearly 14% of households with children.^1^ By May 2020, nearly 18% of US nonelderly adults and 22% of parents with children reported food insecurity in the prior month.^2^ Food insecurity is associated with a wide range of negative health outcomes and with higher healthcare costs.^3^–^11^ Large racial and ethnic inequities exist in food insecurity, as they do for health outcomes broadly; Black and Latinx households are disproportionately affected compared to White households.^2^

A systematic review of the literature on social needs of emergency department (ED) patients found that prevalence of food insecurity is even higher among ED patients than among the general public.^12^ Studies have also found associations between food insecurity and more frequent ED use among specific groups including people experiencing homelessness,^13^,^14^ and people with diabetes,^15^ and among low-income Americans more generally.^16^ Food insecurity may lead to increased ED use due to its association with poor physical and mental health,^11^ worse control of chronic diseases,^11^,^17^,^18^ and medication non-adherence,^19^ which are in turn associated with ED use.^20^–^22^ We build on past literature by examining the association of food insecurity and frequent ED use among a large, random sample of ED patients not restricted to any particular subpopulation. We aimed to increase understanding of potential pathways between food insecurity and frequent ED use by examining whether poor physical and mental health might be mediators of this relationship.

## METHODS

### Study Design

We describe a cross-sectional, secondary analysis of baseline survey questionnaires conducted with randomly sampled ED patients as part of a larger study described previously.^23^,^24^ The study was approved by the NYU School of Medicine Institutional Review Board.

### Setting and Participants

Data collection occurred at a large, urban, public hospital in New York City from November 2016–September 2017. Adult (≥18 years) ED patients were eligible if they were medically/psychiatrically stable, not in prison/police custody, and spoke English or Spanish. Research assistant (RA) shift schedules rotated over time to cover all hours of the day and days of the week, with the number of shifts scheduled during a given time window over the course of the study approximately mirroring ED patient arrival volumes. The RAs approached patients following a random sampling scheme; they then read questions aloud and recorded responses using REDCap (Vanderbilt University, Nashville, TN) secure electronic data capture tools.^25^ Participants provided written informed consent and received $15 compensation.

**Population Health Research Capsule**

What do we already know about this issue?

*Emergency department (ED) patients have a high prevalence of social needs including food insecurity. Associations of food insecurity with frequent ED use are not well documented*.

What was the research question?

*Is food insecurity associated with self-reported frequent ED use in a sample of public hospital ED patients?*

What was the major finding of the study?

*Food insecurity was prevalent among our patient sample and was significantly associated with frequent ED use*.

How does this improve population health?

*Future interventions targeted to frequent ED users should address the high prevalence of unmet social needs such as food insecurity in this population*.

### Measures

Measures were self-reported. We defined frequent ED use as self-report of ≥4 ED visits in the past 12 months, to any ED, including the current visit. While there is no standard definition of “frequent ED use,” ≥4 visits within one year is commonly used.^26^ Past research has found patients self-report ED visits with good accuracy.^27^

Participants answered four questions about food insecurity in the past 12 months from the widely used US Department of Agriculture (USDA) Household Food Security Survey.^28^ Questions were as follows: 1) I/we worried whether my/our food would run out before I/we got money to buy more; 2) The food that (I/we) bought just didn’t last, and (I/we) didn’t have money to get more; 3) (I/we) couldn’t afford to eat balanced meals; and 4) Did you ever eat less than you felt you should because there wasn’t enough money for food?

For the first three questions, participants responded “never true,” “sometimes true,” or “often true.” For the last question—a measure that identifies *very low food security*^1^ (sometimes called *food insufficiency*)—participants responded “yes” or “no,” with yes being considered an affirmative response. The primary independent variable was *any food insecurity*, defined as participants giving an affirmative response (both “sometimes true” or “often true” were included as affirmative) to any of the four items. We separately examined association of frequent ED use with only the more severe form of food insecurity, food insufficiency. In bivariate analyses we also examined each food insecurity question separately and the number (0–4) of questions answered affirmatively.

Covariates included age, gender, race and ethnicity, insurance status, difficulty meeting essential expenses (past year),^29^ homelessness (living in a shelter, unsheltered, or doubled up; past year), unhealthy alcohol use,^30^ and moderate or greater problems with drug use as measured by the Drug Abuse Screening Test (DAST-10). We decided a priori to examine physical health, anxiety, and depression as potential mediators based on prior literature and theory positing these factors as sensitive to food insecurity and as strong drivers of ED use.^11^,^22^,^31^,^32^ Self-reported overall health was measured using a single item from the US Centers for Disease Control and Prevention Health-Related Quality of Life score, asking “Would you say that in general your health is”; possible answers were excellent, very good, good, fair, and poor.^33^ Anxiety was measured using the GAD-2 (general anxiety disorder) and depression using the PHQ-2 (patient health questionnaire); both are previously validated two-item screeners.^34^,^35^

### Analysis

We examined bivariate associations using chi-squared tests of independence for categorical variables. We conducted multivariable logistic regression to examine the independent association of any food insecurity and food insufficiency with frequent ED use while adjusting for potential confounders. As described above, we examined for mediation by anxiety or depression and overall self-rated health. Anxiety and depression were combined into a single binary variable due to significant collinearity; no other variables demonstrated significant collinearity (Spearman correlation coefficients < 0.4). We examined mediation by including the hypothesized mediators in adjusted regression models and determining whether effect estimates were attenuated compared to effect estimates in models including confounders but without hypothesized mediators.^36^ Complete case deletion was used in regression models; the amount of missing data was small (3.7%).

## RESULTS

Research assistants approached 6097 patients, of whom 2924 (48%) were eligible. The most common reasons for ineligibility were being medically unstable, intoxicated, not speaking English/Spanish, or in police/prison custody. Of eligible patients, 2396 (82%) agreed to participate. After removing duplicate records for patients who participated more than once (n = 84) there were 2312 participants. Three did not answer the question about past ED use (n = 3) and were excluded from bivariate and multivariable analyses. Participants were diverse in gender, race and ethnicity, and age (Table 1). Half (50.8%) reported any food insecurity. Many also reported difficulty meeting basic expenses and past year homelessness. Nearly one-third (30.9%) were frequent ED users.

Participants who reported frequent ED use had significantly higher prevalence of food insecurity than other ED patients (Table 2). This finding held true across each individual food insecurity question and for food insecurity overall, with 62.8% of participants with frequent ED use endorsing any food insecurity vs 45.4% of participants who did not report frequent ED use (*P* <0.001).

In multivariable analyses (Table 3), both any food insecurity (adjusted odds ratio [aOR] 1.48, 95% confidence interval [CI], 1.20–1.83) and food insufficiency (aOR 1.45, 95% CI, 1.16–1.83) were associated with frequent ED use. These relationships were partially attenuated in models adding depression/anxiety and overall health status, with persistently significant yet reduced aORs for the associations of food insecurity/insufficiency and frequent ED in mediation models.

## DISCUSSION

We found a robust association between food insecurity and frequent ED use, including in multivariable analyses adjusting for potential confounders. This relationship was partially attenuated by controlling for anxiety/depression and overall health status, suggesting the possibility of mediation. Notably, while prevalence of food insecurity was highest among participants who reported frequent ED use, even participants without frequent ED use had a high prevalence of food insecurity.

Our findings are consistent with past research showing ED patients have a high prevalence of social needs, including food security. A systematic review^12^ showed that while studies varied, food insecurity prevalence of ED patients was generally above 20%, with several studies finding prevalence of one-third or even higher.^12^ A few studies examining the association of food insecurity and frequent ED use, among specific subgroups^13^–^15^ and more generally,^16^ have uniformly found a significant association. Other studies have found food-insecure ED patients are more likely to have chronic pain, mental health concerns, substance use, and homelessness, all of which are known to be associated with frequent ED use.^3^,^37^

Our study was unique in randomly sampling a large number of ED patients and including multiple measures of food insecurity, as well as examining the independent association of food insecurity with frequent ED use while controlling for possible confounders and exploring mediation. Although we cannot prove causality in this cross-sectional study, one potential hypothesis is that food insecurity contributes to anxiety/depression and poor overall health, which in turn contributes to frequent ED use. This hypothesis could be examined in future longitudinal research.

The strong association observed between food insecurity and frequent ED use in this study has implications for programs aiming to reduce frequent ED use. Frequent ED use has been the subject of persistent programmatic and policy attention in the US, although programs to address it have had variable success, particularly when examined using robust study designs.^38^ Our study adds to evidence suggesting the importance of assessing and addressing the social and structural conditions of people’s lives as an integral part of programs developed to reduce frequent ED use.

There has been increased interest nationally in screening for patient social needs in healthcare settings. For food insecurity, a two-item screening tool called the Hunger Vital Sign has been well tested and validated in healthcare settings.^39^–^42^ The items are based on two of the USDA Food Security Survey questions used in our study, on worry about food running out and food not lasting. Research indicates patients generally feel positively about being asked such questions in healthcare settings, including EDs.^43^,^44^ Some studies have suggested patients may prefer and more readily disclose food insecurity when electronic tablet-based screening is used,^45^ although other studies have suggested no difference in social needs disclosure with tablet vs in-person interviews.^46^

To date, few studies have rigorously examined how to best assist ED patients who screen positive for food insecurity. A systematic review by De Marchis et al found 23 studies—most of which were of low quality—that examined interventions addressing food insecurity in healthcare settings.^47^ One study found having an electronic health record order for referral to a local food bank partner (with patient contact information sent to the food bank and the food bank proactively contacting patients) resulted in more ED patients receiving referrals, 63% of whom ultimately received assistance.^48^

## LIMITATIONS

Our study results should be interpreted in light of a few limitations. First, measures were self-reported. We used validated questions when available and chose measures for which we expected self-report to be accurate. Second, this study was conducted in a single public hospital ED serving a patient population with high levels of social needs. However, multiple other studies conducted in geographically diverse EDs have found that ED patients have a high prevalence of food insecurity.^12^ Even if prevalence of food insecurity and other participant characteristics in our study differed from those of patients at other EDs, we do not expect the relationship between food insecurity and frequent ED use would be unique to the ED patients we studied.

Finally, we cannot suggest causality for relationships observed in this cross-sectional study. Although we controlled for multiple potential confounders, including other measures of socioeconomic status, there is a possibility of unmeasured confounders. Additionally, although we postulate one hypothetical causal pathway in our mediation analyses, the data remain cross-sectional; we were unable to prove causal associations, and mental health and health status could potentially be confounders as well as mediators. Additional longitudinal and qualitative research could further elucidate the relationship of food insecurity and frequent ED use. We also suggest implementing and studying programs to assist ED patients with food insecurity.

## CONCLUSION

We found a high prevalence of food insecurity among ED patients in our study population. Food insecurity was significantly associated with frequent ED use. Efforts to improve care of patients who frequently visit the ED should assess and address social needs including food insecurity; even apart from any potential effects on reducing future ED use, having adequate food is a critical human need that such efforts could be well-positioned to help address. More generally, EDs have long been described as “social welfare institutions,”^49^ and there has been a recent resurgence of interest within emergency medicine in patient social needs.^50^ This study adds to the body of evidence supporting the potentially important role of EDs in assisting patients with food insecurity.

## Figures and Tables

**Table 1:** Participant characteristics.

	n (%)^a^ n=2,312
Sociodemographics	
Age	
18–30	488 (21.1)
31–50	855 (37.0)
51–65	689 (29.8)
>65	279 (12.1)
Gender	
Female	1,006 (43.8)
Male	1,293 (56.2)
Race/ethnicity	
Hispanic/Latino	1,270 (55.3)
Non-Hispanic Black	531 (23.1)
Non-Hispanic White	280 (12.2)
Other	217 (9.4)
Insurance	
Uninsured	621 (26.9)
Medicaid and/or Medicare	1,202 (52.1)
Private / Other	485 (21.0)
Unable to meet essential expenses, past 12 months	936 (40.8)
Homelessness (including living doubled up^b^, past 12 months)	492 (21.4)
Health	
Number of ED visits, past 12 months (including current visit)	
1	754 (32.6)
2	466 (20.2)
3	375 (16.2)
4+	714 (30.9)
Overall self-rated health	
Excellent or very good	538 (23.4)
Good	722 (31.4)
Fair	754 (32.8)
Poor	287 (12.5)
Moderate or greater problems with drug use (by DAST-10)	276 (12.0)
Unhealthy alcohol use	747 (32.4)
Positive screen for anxiety (GAD-2) or depression (PHQ-2)	859 (37.6)
Food insecurity (past 12 months)	
Worried food would run out before got money to buy more	
Often true	299 (13.1)
Sometimes true	586 (25.6)
Never true	1,403 (61.3)
Food didn’t last and didn’t have money to get more	
Often true	264 (11.5)
Sometimes true	558 (24.4)
Never true	1,466 (64.1)
Couldn’t afford to eat balanced meals	
Often true	284 (12.4)
Sometimes true	562 (24.6)
Never true	1,437 (62.9)
Ate less than felt should because not enough money for food (yes)	632 (27.7)
Any food insecurity (any of 4 questions answered affirmatively)	1,159 (50.8)
Number of food insecurity questions answered affirmatively	
0	1,122 (49.3)
1	245 (10.8)
2	220 (9.7)
3	260 (11.4)
4	427 (18.8)

a Percentages shown are among those who answered a given question; denominators for some questions are <2,312 due to a small amount of missing data for some questions (never exceeding 1.6%).

b Living “doubled up” includes “couch surfing” or staying with friends, family members, or others due to lack of other housing options.

*ED*, emergency department; *DAST*, drug abuse screening test; *GAD*, generalized anxiety disorder; *PHQ*, patient health questionnaire.

**Table 2:** Food insecurity and other characteristics for patients by frequent emergency department (ED) use status.

	Frequent ED Use n (%) n=714^a^	No Frequent ED Use n (%) n=1595^a^	*P*-value^b^
Sociodemographics			
Age			<0.001
18–30	122 (17.1)	366 (22.9)	
31–50	246 (34.5)	609 (38.2)	
51–65	257 (36.0)	429 (26.9)	
>65	88 (12.3)	191 (12.0)	
Gender			0.02
Female	285 (40.1)	719 (45.4)	
Male	426 (59.9)	866 (54.6)	
Race/ethnicity			<0.001
Hispanic/Latino	348 (49.1)	920 (58.0)	
Non-Hispanic Black	219 (30.9)	311 (19.6)	
Non-Hispanic White	80 (11.3)	200 (12.6)	
Other	62 (8.7)	155 (9.8)	
Insurance			<0.001
Uninsured	122 (17.1)	499 (31.3)	
Medicaid and/or Medicare	454 (63.7)	746 (46.8)	
Private / Other	137 (19.2)	348 (21.8)	
Unable to meet essential expenses, past 12 months	365 (51.5)	571 (36.1)	<0.001
Homelessness (including doubled up), past 12 months	256 (36.1)	235 (14.8)	<0.001
Health			
Overall self-rated health			<0.001
Excellent or very good	112 (15.8)	426 (26.7)	
Good	182 (25.7)	540 (33.9)	
Fair	262 (37.1)	491 (30.8)	
Poor	151 (21.4)	136 (8.5)	
Moderate or greater problems with drug use	130 (18.4)	146 (9.2)	<0.001
Unhealthy alcohol use	247 (34.8)	500 (31.4)	0.11
Positive screen for anxiety or depression	362 (51.5)	496 (31.4)	<0.001
Food insecurity (past 12 months)			
Worried food would run out before got money to buy more			<0.001
Often true	141 (19.9)	157 (9.9)	
Sometimes true	205 (28.9)	381 (24.1)	
Never true	363 (51.2)	1040 (65.9)	
Food didn’t last and didn’t have money to get more			<0.001
Often true	140 (19.8)	124 (7.8)	
Sometimes true	201 (28.4)	356 (22.5)	
Never true	366 (51.8)	1, 100 (69.6)	
Couldn’t afford to eat balanced meals			<0.001
Often true	149 (21.2)	135 (8.6)	
Sometimes true	196 (27.8)	365 (23.1)	
Never true	359 (60.0)	1078 (68.3)	
Ate less than should because not enough money	276 (39.1)	355 (22.5)	<0.001
Any food insecurity	444 (62.8)	714 (45.4)	<0.001
Food insecurity questions answered affirmatively			<0.001
0	263 (37.5)	859 (54.7)	
1	73 (10.4)	172 (10.9)	
2	68 (9.7)	152 (9.7)	
3	103 (14.7)	157 (10.0)	
4	195 (27.8)	231 (14.7)	

a Percentages shown are among those who answered a given question; denominators for some questions are lower due to a small amount of missing data for some questions (never exceeding 1.7%).

b P-values for bivariate associations tested using chi-squared tests of independence for categorical variables.

**Table 3:** Association of emergency department (ED) patient food insecurity with frequent ED use.

	Unadjusted Model OR (95% CI)	Adjusted Model^a^ OR (95% CI)	Mediation Model^b^ OR (95% CI)
Any food insecurity^c^	2.03 (1.69–2.44)	1.48 (1.20–1.83)	1.36 (1.09–1.70)^e^
Food insufficiency^d^	2.21 (1.82–2.67)	1.45 (1.16–1.83)	1.29 (1.02–1.64)^e^

a Adjusted models include: gender, race and ethnicity, age category, insurance status, homelessness in past 12 months, difficulty meeting essential expenses in past 12 months, unhealthy alcohol use, and moderate or greater drug use problems.

b Mediation models additionally adjust for anxiety/depression (positive GAD-2 or PHQ-2) and self-rated overall health. Independent effect estimates for the association between the mediators and outcomes in the mediation models were all positive and statistically significant (not shown).

c Any food insecurity defined as affirmative response (often true or sometimes true coded as affirmative) to ≥1 of the 4 US Department of Agriculture (USDA) food insecurity questions asked (over the past 12 months).

d Food insufficiency defined as a “yes” response to USDA question of whether participant had eaten less than they felt they should because there was not enough money for food in the past 12 months.

e Parameter estimates for any food insecurity and food insufficiency reduced in mediation models by 21.2% and 31.5%, respectively.

*OR*, odds ratio; *CI*, confidence interval.

## References

[1] USDA Economic Research Service (2020). Key Statistics & Graphics. Food Security Status of U.S. Households in 2019.

[2] Waxman E, Gupta P, Karpman M (2020). More than one in six adults were food insecure two months into the COVID-19 recession: findings from the May 14–27 Coronavirus Tracking Survey.

[3] Sullivan AF, Clark S, Pallin DJ (2010). Food security, health, and medication expenditures of emergency department patients. J Emerg Med.

[4] Montgomery J, Lu J, Ratliff S (2017). Food insecurity and depression among adults with diabetes: results from the National Health and Nutrition Examination Survey (NHANES). Diabetes Educ.

[5] Silverman JB, Krieger J, Kiefer MM (2014). The association of food insecurity and diabetes control among low-income individuals. J Gen Intern Med.

[6] Pan L, Sherry B, Njai R (2012). Food insecurity is associated with obesity among US adults in 12 states. J Acad Nutr Diet.

[7] Berkowitz SA, Seligman HK, Choudhry NK (2014). Treat or eat: food insecurity, cost-related medication underuse, and unmet needs. Am J Med.

[8] Knight CK, Probst JC, Liese AD (2016). Household food insecurity and medication “scrimping” among US adults with diabetes. Prev Med.

[9] Berkowitz SA, Seligman HK, Meigs JB (2018). Food insecurity, healthcare utilization, and high cost: a longitudinal cohort study. Am J Manag Care.

[10] Berkowitz SA, Basu S, Gundersen C (2019). State-level and county-level estimates of health care costs associated with food insecurity. Prev Chronic Dis.

[11] Gundersen C, Ziliak JP (2015). Food insecurity and health outcomes. Health Aff (Millwood).

[12] Malecha PW, Williams JH, Kunzler NM (2018). Material needs of emergency department patients: a systematic review. Acad Emerg Med.

[13] Weiser SD, Hatcher A, Frongillo EA (2013). Food insecurity is associated with greater acute care utilization among HIV-infected homeless and marginally housed individuals in San Francisco. J Gen Intern Med.

[14] Baggett TP, Singer DE, Rao SR (2011). Food insufficiency and health services utilization in a national sample of homeless adults. J Gen Intern Med.

[15] Becerra MB, Allen NL, Becerra BJ (2016). Food insecurity and low self-efficacy are associated with increased healthcare utilization among adults with type II diabetes mellitus. J Diabetes Complications.

[16] Kushel MB, Gupta R, Gee L (2006). Housing instability and food insecurity as barriers to health care among low-income Americans. J Gen Intern Med.

[17] Seligman HK, Jacobs EA, López A (2012). Food insecurity and glycemic control among low-income patients with type 2 diabetes. Diabetes Care.

[18] Schroeder EB, Zeng C, Sterrett AT (2019). The longitudinal relationship between food insecurity in older adults with diabetes and emergency department visits, hospitalizations, hemoglobin A1c, and medication adherence. J Diabetes Complications.

[19] Caouette S, Boss L, Lynn M (2020). The relationship between food insecurity and cost-related medication nonadherence in older adults: a systematic review. Am J Nurs.

[20] Blanchard J, Madden JM, Ross-Degnan D (2013). The relationship between emergency department use and cost-related medication nonadherence among Medicare beneficiaries. Ann Emerg Med.

[21] Doran KM, Shumway M, Hoff RA (2014). Correlates of hospital use in homeless and unstably housed women: the role of physical health and pain. Womens Health Issues.

[22] Doupe MB, Palatnick W, Day S (2012). Frequent users of emergency departments: developing standard definitions and defining prominent risk factors. Ann Emerg Med.

[23] Doran KM, Rahai N, McCormack RP (2018). Substance use and homelessness among emergency department patients. Drug Alcohol Depen.

[24] Gerber E, Gelberg L, Rotrosen J (2020). Health-related material needs and substance use among emergency department patients. Subst Abus.

[25] Harris PA, Taylor R, Thielke R (2009). Research electronic data capture (REDCap)--a metadata-driven methodology and workflow process for providing translational research informatics support. J Biomed Inform.

[26] LaCalle E, Rabin E (2010). Frequent users of emergency departments: the myths, the data, and the policy implications. Ann Emerg Med.

[27] Hwang SW, Chambers C, Katic M (2016). Accuracy of self-reported health care use in a population-based sample of homeless adults. Health Serv Res.

[28] U.S. Department of Agriculture USDA Food Security Survey.

[29] U.S. Census Bureau Survey of Income and Program Participation.

[30] Saitz R, Cheng DM, Allensworth-Davies D (2014). The ability of single screening questions for unhealthy alcohol and other drug use to identify substance dependence in primary care. J Stud Alcohol Drugs.

[31] Doran KM, Raven MC, Rosenheck RA (2013). What drives frequent emergency department use in an integrated health system? National data From the Veterans Health Administration. Ann Emerg Med.

[32] Gelberg L, Andersen RM, Leake BD (2000). The Behavioral Model for Vulnerable Populations: application to medical care use and outcomes for homeless people. Health Serv Res.

[33] Centers for Disease Control and Prevention CDC HRQOL-4 ‘Healthy Days Measure’.

[34] Plummer F, Manea L, Trepel D (2016). Screening for anxiety disorders with the GAD-7 and GAD-2: a systematic review and diagnostic metaanalysis. Gen Hosp Psychiatry.

[35] Kroenke K, Spitzer RL, Williams JB (2003). The Patient Health Questionnaire-2: validity of a two-item depression screener. Med Care.

[36] Baron RM, Kenny DA (1986). The moderator-mediator variable distinction in social psychological research: conceptual, strategic, and statistical considerations. J Pers Soc Psychol.

[37] Miner JR, Westgard B, Olives TD (2013). Hunger and food insecurity among patients in an urban emergency department. West J Emerg Med.

[38] Althaus F, Paroz S, Hugli O (2011). Effectiveness of interventions targeting frequent users of emergency departments: a systematic review. Ann Emerg Med.

[39] Makelarski JA, Abramsohn E, Benjamin JH (2017). Diagnostic accuracy of two food insecurity screeners recommended for use in health care settings. Am J Public Health.

[40] Gattu RK, Paik G, Wang Y (2019). The Hunger Vital Sign identifies household food insecurity among children in emergency departments and primary care. Children (Basel).

[41] Hager ER, Quigg AM, Black MM (2010). Development and validity of a 2-item screen to identify families at risk for food insecurity. Pediatrics.

[42] Gundersen C, Engelhard EE, Crumbaugh AS (2017). Brief assessment of food insecurity accurately identifies high-risk US adults. Public Health Nutr.

[43] De Marchis EH, Hessler D, Fichtenberg C (2019). Part I: a quantitative study of social risk screening acceptability in patients and caregivers. Am J Prev Med.

[44] Byhoff E, De Marchis EH, Hessler D (2019). Part II: a qualitative study of social risk screening acceptability in patients and caregivers. Am J Prev Med.

[45] Cullen D, Woodford A, Fein J (2019). Food for thought: a randomized trial of food insecurity screening in the emergency department. Acad Pediatr.

[46] Gottlieb L, Hessler D, Long D, Amaya A, Adler N (2014). A randomized trial on screening for social determinants of health: the iScreen study. Pediatrics.

[47] De Marchis EH, Torres JM, Benesch T (2019). Interventions addressing food insecurity in health care settings: a systematic review. Ann Fam Med.

[48] Martel ML, Klein LR, Hager KA (2018). Emergency department experience with novel electronic medical record order for referral to food resources. West J Emerg Med.

[49] Gordon JA (1999). The hospital emergency department as a social welfare institution. Ann Emerg Med.

[50] Anderson ES, Lippert S, Newberry J (2016). Addressing social determinants of health from the emergency department through social emergency medicine. West J Emerg Med.

